# Defining the optimal spirometry cut-off points for optimal bronchodilator response in prematurity-associated lung disease

**DOI:** 10.1183/23120541.00239-2026

**Published:** 2026-09-01

**Authors:** John Lowe, Michael Cousins, Kylie Hart, Sarah J. Kotecha, W. John Watkins, Sailesh Kotecha

**Affiliations:** 1 Centre for Trials Research, College of Biomedical and Life Sciences, Cardiff University, Cardiff, UK; 2 Department of Child Health, Cardiff University School of Medicine, Cardiff, UK

## Abstract

It is increasingly accepted that dysfunctional spirometry is associated with preterm birth, a disease process termed prematurity-associated lung disease (PLD) [1]. It is also accepted that the treatment of PLD is uncertain, with two recent trials showing no significant improvements after 12 weeks of inhaled corticosteroids [2]. The trial by Goulden *et al.* [2] suggested improvements after a combination of inhaled corticosteroids and a long-acting β_2_-bronchodilator.

## Shareable abstract

**The best approach to identification and management of prematurity-associated lung disease is uncertain. This study describes a method for identification of at-risk children who may be amenable to inhaled therapy.** https://bit.ly/4vvvyR9

*To the Editor:*

It is increasingly accepted that dysfunctional spirometry is associated with preterm birth, a disease process termed prematurity-associated lung disease (PLD) [1]. It is also accepted that the treatment of PLD is uncertain, with two recent trials showing no significant improvements after 12 weeks of inhaled corticosteroids [2]. The trial by Goulden *et al.* [2] suggested improvements after a combination of inhaled corticosteroids and a long-acting β_2_-bronchodilator. However, in preterm-born children, the optimal values to identify optimal bronchodilator response are poorly documented. The European Respiratory Society (ERS) recommendations of 12% improvement to determine bronchial reversibility is far too generous for the preterm-born population especially when it is noted that those with bronchopulmonary dysplasia (BPD) have deficits in mean % predicted forced expiratory volume in 1 s (FEV_1_) of ∼16% less than term controls in a recent systematic review [1]. Even a smaller absolute improvement of 10% FEV_1_ % predicted after bronchodilator (as recommended by the American Thoracic Society (ATS)) is likely to result in significant clinical improvements. Therefore, in a large preterm-born population, we aimed to identify optimal spirometry values to identify children most likely to respond to bronchodilators.

The Respiratory Health Outcomes in Neonates (RHiNO) study (EudraCT identifier 2015-003712-20) has been described previously [2]. Briefly, children born at ≤34 weeks’ gestation aged between 7 and 12 years were invited for a home or hospital visit to obtain measurement of spirometry (Microloop; CareFusion, Wokingham, UK) before and after administration of a bronchodilator (4×100-μg puffs of salbutamol administered *via* a spacer device). Spirometry was performed and quality controlled as per the ERS/ATS guidelines [3], and normalised against Global Lung Function Initiative reference values [4]. Recruitment took place prospectively between November 2016 and September 2019.

Differences in baseline characteristics between groups (bronchodilator responders *versus* nonresponders) were compared with t-tests and Chi-squared tests for continuous variables and categorical variables, respectively. Area under the receiver operating characteristic curve (AUROC) analysis was undertaken to assess the diagnostic value of baseline spirometry measures to identify a binary outcome: those who had a positive bronchodilator reversibility (>10% change in FEV_1_ % predicted after bronchodilation) and those who did not. Youden's J-statistic was utilised to identify the cut-off value with the optimal balance between sensitivity and specificity levels. Data analyses were performed using SPSS version 26 (IBM, Armonk, NY, USA).

Following quality control checks, both pre-and post-bronchodilator spirometry data were available from 516 (95%) out of 544 preterm-born children. Baseline characteristics comparing those who responded to bronchodilator with those who did not are presented in table 1. 66 (13%) out of 516 responded to salbutamol with an FEV_1_ % predicted increase of ≥10%. It was notable that, despite having bronchodilator response, only 40% had received inhalers over the previous year, suggesting marked undertreatment for airway obstruction. Mean values for baseline FEV_1_ % predicted, forced vital capacity (FVC) % predicted and FEV_1_/FVC ratio were all lower in the responder group. Responders also had a higher rate of diagnosis of BPD in infancy (30% *versus* 19%), reported more wheeze ever (71% *versus* 52%), recent wheeze (49% *versus* 26%) and had increased inhaler use (40% *versus* 15%). Exhaled nitric oxide fraction (*F*_ENO_) was increased in the bronchodilator responsive group (mean±sd 30.5±26.2 ppb) when compared with the nonresponsive group (16.0±16.4 ppb).

**TABLE 1:** Baseline characteristics and optimal cut-offs

Characteristic	No BDR	BDR	Total	p-value
**Subjects**	450	66	516	
**Males**	227 (50)	34 (52)	261 (51)	ns
**Age, years, mean (range)**	9.7 (7–12)	9.4 (7–12)	9.6 (7–12)	ns
**Gestational age, weeks, mean** **(range)**	31 (23–34)	30 (24–34)	31 (23–34)	ns
**Birthweight, g, median (IQR)**	1700 (1276–2070)	1662 (995–2232)	1700 (1219–2098)	ns
**Bronchopulmonary dysplasia**	85 (19)	20 (30)	105 (20)	0.03
**Wheeze ever** ^#^	222/430 (52)	46/65 (71)	268/495 (54)	0.004
**Recent^¶^ wheeze**	115 (26)	32 (49)	147 (28)	<0.001
**Diagnosed asthma** ^#^	61/449 (14)	25/66 (38)	86/515	<0.001
**Inhalers in last 12 months**	67 (15)	25 (40)	92 (18)	<0.001
**FEV_1_, % predicted**	92.9±11.4	80.0±15.0	91.2±12.7	<0.001
**FEF_25–75%_, % predicted**	80.0±19.1	55.1±18.7	76.6±20.8	<0.001
**FVC, % predicted**	94.9±11.1	91.6±11.9	94.5±11.3	0.04
**FEV_1_/FVC**	0.86±0.07	0.75±0.10	0.84±0.08	<0.001

Parameter	Sensitivity	Specificity	Positive predictive value	Negative predictive value	Youden's J-statistic
**FEV_1_**					
70% predicted	0.976	0.258	0.900	0.607	0.23
75% predicted	0.938	0.318	0.904	0.429	0.26
80% predicted	0.880	0.439	0.915	0.349	0.32
**85% predicted**	**0.789**	**0.591**	**0.929**	**0.291**	**0.38**
90% predicted	0.591	0.773	0.947	0.217	0.36
z-score^+^	0.802	0.591	0.93	0.305	0.39
LLN	0.876	0.455	0.916	0.349	0.33
**FEF_25–75%_**					
60% predicted	0.842	0.652	0.943	0.377	0.49
**65% predicted**	**0.773**	**0.727**	**0.951**	**0.320**	**0.50**
70% predicted	0.667	0.803	0.958	0.261	0.47
75% predicted	0.582	0.879	0.97	0.236	0.46
80% predicted	0.491	0.939	0.982	0.213	0.43
z-score^+^	0.704	0.803	0.961	0.285	0.51
LLN	0.771	0.712	0.948	0.313	0.48
**FVC**					
70% predicted	0.982	0.03	0.874	0.200	0.012
75% predicted	0.964	0.091	0.879	0.273	0.055
**80% predicted**	**0.911**	**0.212**	**0.887**	**0.259**	**0.12**
85% predicted	0.809	0.303	0.888	0.189	0.11
90% predicted	0.658	0.455	0.892	0.163	0.11
z-score^+^	0.896	0.288	0.896	0.288	0.18
LLN	0.902	0.242	0.89	0.267	0.14
**FEV_1_/FVC**					
0.70	0.978	0.212	0.894	0.583	0.19
0.75	0.942	0.455	0.922	0.536	0.40
**0.80**	**0.816**	**0.697**	**0.948**	**0.357**	**0.51**
0.85	0.556	0.864	0.965	0.222	0.42
z-score^+^	0.813	0.712	0.951	0.359	0.53
LLN	0.911	0.545	0.932	0.474	0.46

Data are presented as n (%), n/N (%) or mean±sd, unless otherwise stated. Rows in bold represent optimal cut-offs. BDR: bronchodilator reversibility; IQR: interquartile range; FEV_1_: forced expiratory volume in 1 s; FEF_25–75%_: forced expiratory flow at 25–75% forced vital capacity; FVC: forced vital capacity. ns: not significant; LLN: lower limit of normal. ^#^: denominators differ due to missing questionnaire data; ^¶^: <12 months; ^+^: best Youden's J-statistic.

AUROC was 0.758, 0.825 and 0.816 for FEV_1_ % predicted, forced expiratory flows between 25–75% FVC (FEF_25–75%_) % predicted and FEV_1_/FVC ratio, respectively, but was 0.576 for FVC % predicted. The spirometry cut-off values for optimal Youden's J-statistic values were 85% for FEV_1_ % predicted (sensitivity 0.789 and specificity 0.591), 65% for FEF_25–75%_ % predicted (sensitivity 0.773 and specificity 0.727) and 0.80 for FEV_1_/FVC (sensitivity 0.816 and specificity 0.697). Respective optimal Youden's J-statistic values when using the lower limit of normal (LLN) as a cut-off were lower at 0.33 for FEV_1_ (sensitivity 0.876 and specificity 0.455), 0.48 for FEF_25–75%_ (sensitivity 0.771 and specificity 0.712) and 0.46 for FEV_1_/FVC ratio (sensitivity 0.911 and specificity 0.545), thus had marginally greater sensitivity but at the expense of lesser specificity than for the % predicted values identified above. The optimal Youden's J-statistic values were similar to % predicted values for all measurements when z-scores were used (table 1).

Finally, we classified the preterm-born children as PLD or preterm controls using spirometry criteria reported recently [5, 6]. 119 (23%) were noted to have PLD and 397 (77%) were classified as preterm controls. 43 (36.1%) out of 119 and 23 (5.8%) out of 397 from the PLD and preterm groups, respectively, had bronchodilator responses, giving an odds ratio of 9.20 (95% CI 5.24–16.21, p<0.0001).

By studying a large group of preterm-born children, we were able to identify optimal cut-off values for spirometry measures to identify children who would best to respond to bronchodilators. The vast majority of the children with bronchodilator responses were from the recently defined PLD group. The values identified within the AUROC analysis for both FEV_1_ (85.5% predicted) and FEV_1_/FVC (0.79) are very similar to previously utilised pragmatically chosen cut-off levels to characterise obstructive and nonobstructive respiratory disease [2], thus suggesting that the use of either of these cut-offs for baseline spirometry might more accurately identify those who would benefit from inhaled therapy. Although the highest AUROC was associated with FEF_25–75%_ % predicted, this measure is associated with great variability, especially as it is associated with the lower end of the forced expiry curve [7].

The recent ERS Task Force and ATS reports on the management of BPD after discharge from the neonatal unit could not identify any evidence-based studies to recommend the use of either inhaled corticosteroids or bronchodilators [8, 9]. Even less has been reported for preterm-born children who have low lung function but do not have BPD in infancy. Identification of children with PLD who may respond is clearly especially important as only 40% had received inhalers over the previous year despite having significant bronchodilator response. This potential undertreatment is likely attributable to the lack of a diagnostic label and the absence of follow-up beyond infancy. Given the recent data showing improvement after combined therapy [2], it would be reasonable to improve this marked undertreatment with a 12-week trial of combined inhaled corticosteroid and long-acting bronchodilators, and to continue or stop depending on whether a response is noted. There are no satisfactory cut-off values for children and the LLN is associated with better sensitivity but poor specificity and does not appear to be useful for identifying children who respond to bronchodilators; thus, using a clinically useful cut-off for optimal bronchodilator response for preterm-born children with PLD is reasonable. *F*_ENO_ was increased in the bronchodilator responsive group but we have not noted any atopic association with PLD, as previously published [10]. *F*_ENO_ has the potential for both identification of PLD and in monitoring response to treatment [2]. We have published lung volumes, imaging with hyperpolarised xenon scanning and lung clearance index for children with and without PLD [5, 10, 11]. Thus, the identified cut-off values can be utilised in community screening to support referrals for these in-depth investigations.

The main strength of this study is that we have investigated one of the largest groups of preterm-born children with standardised assessments. We acknowledge that the ERS criterion for reversibility in children is a ≥12% change from baseline; however, these guidelines are for identification of asthma rather than PLD. We view 10% to be adequate as an initial screening tool especially given the far lower spirometry associated with extremely prematurity, whereby even a small increase in lung function is likely to be associated with improved exercise capacity.

We conclude that FEV_1_ 85% predicted and FEV_1_/FVC 0.80 are optimal measures to identify bronchodilator response in children with PLD. Further use of spirometry to classify children into differing respiratory phenotypes may help identify those who will benefit from inhaled treatments following further detailed investigations [5].
